# Associations of self-reported sleep disturbances, sleep onset, and duration with gallstone disease risk

**DOI:** 10.3389/fnut.2025.1593720

**Published:** 2025-06-24

**Authors:** Dongjun Bao, Kunming Bao, Xiaoxian Fu, Xueping Xie, Xin Ma, Zhidong Huang, Rongcai Que, Wenjun Gu, Shengyou Lu

**Affiliations:** ^1^The Second Hospital of Longyan City, Longyan, China; ^2^Longyan First Affiliated Hospital of Fujian Medical University, Longyan, China; ^3^Guangdong Pharmaceutical University, Guangzhou, China; ^4^Huizhou Third People's Hospital, Guangzhou Medical University, Huizhou, China

**Keywords:** trouble sleeping, sleep onset time, sleep duration, gallstone disease, NHANES

## Abstract

**Background:**

The role of sleep disturbances in gallstone disease risk remains unclear. We aimed to examine the associations between sleep disturbances and gallstone disease risk.

**Methods:**

We analyzed data from 9,059 participants in the NHANES survey (2017–2020). The primary outcome of this study was gallstone disease. Sleep disturbances included trouble sleeping, early or late sleep onset time, and long or short sleep duration. Multivariate logistic regression analysis was employed to evaluate the associations between sleep disturbances and gallstone disease risk.

**Results:**

After adjusting for confounding factors, trouble sleeping was associated with an elevated risk of gallstone disease, with the odds ratio (OR) of 1.47 (95% confidence interval [CI]: 1.01–2.15), compared to those without trouble sleeping. In further stratified analysis, among individuals with trouble sleeping, no significant associations were found between different sleep onset intervals, sleep duration and gallstone disease risk. Among participants without trouble sleeping, the sleep onset interval of 23:00 to 00:00 was associated with a significantly increased gallstone disease risk compared to the reference sleep onset interval of 22:00–23:00, with an OR of 1.61 (95% CI: 1.06–2.45). Short sleep duration (<6 h) was associated with a significantly reduced gallstone disease risk compared to the reference sleep duration of 6–8 h, with the OR of 0.43 (95% CI: 0.25–0.75).

**Conclusion:**

This study demonstrates that trouble sleeping increases the risk of gallstone formation, independent of sleep onset time and sleep duration. Among those without trouble sleeping, a sleep onset time between 23:00–00:00 is associated with a higher risk, while short sleep duration (< 6 h) appears protective.

## 1 Introduction

Gallstone disease remains one of the most popular digestive system disorders in the US, influencing approximately 10 to 15% of American adults (1, 2). Gallstone disease often presents asymptomatic in the early stages. Without timely screening and intervention, it may develop symptomatic and result in more serious complications, including cholecystitis, cholangitis, biliary pancreatitis, and even gallbladder and biliary tract cancers, which lead to the worst prognosis (3). In 2015 alone, 1.5 million people sought medical care for gallstone-related diseases, with associated nationwide expenditures totaling 10.3 billion dollars (1). Identifying risk factors for gallstone disease for early detection and intervention plays an important role in mitigating this heavy public health and economic burden.

Numerous risk factors are well-recognized contributors to gallstone formation, including age, gender, physical activity, family history, and insulin resistance. The role of sleep—a modifiable lifestyle factor—remains underexplored (4, 5). Sleep disturbances, including insomnia, hypersomnia, and irregular sleep duration and timing, are prevalent in the American population (6–8), which may indicate underlying circadian disruption (9). Circadian disorders have been linked to various metabolic diseases, including diabetes (10), obesity (11), and hyperlipidemia (12, 13), all of which are also considered risk factors for gallstone disease (5). Evidence from animal studies and population-based research suggests a link between disrupted circadian rhythms and increased gallstone disease risk (14, 15). yet specific studies exploring this association are sparse.

This study aims to address this gap by examining the link between trouble sleeping and gallstone disease. By conducting a stratified analysis based on whether participants experience trouble sleeping, we explore the potential effects of sleep onset time and duration on gallstone formation. Understanding this link could provide valuable insights into novel preventative and intervention strategies, highlighting the importance of sleep health in gallstone disease prevention.

## 2 Methods

### 2.1 Data source and study population

**Figure 1:**
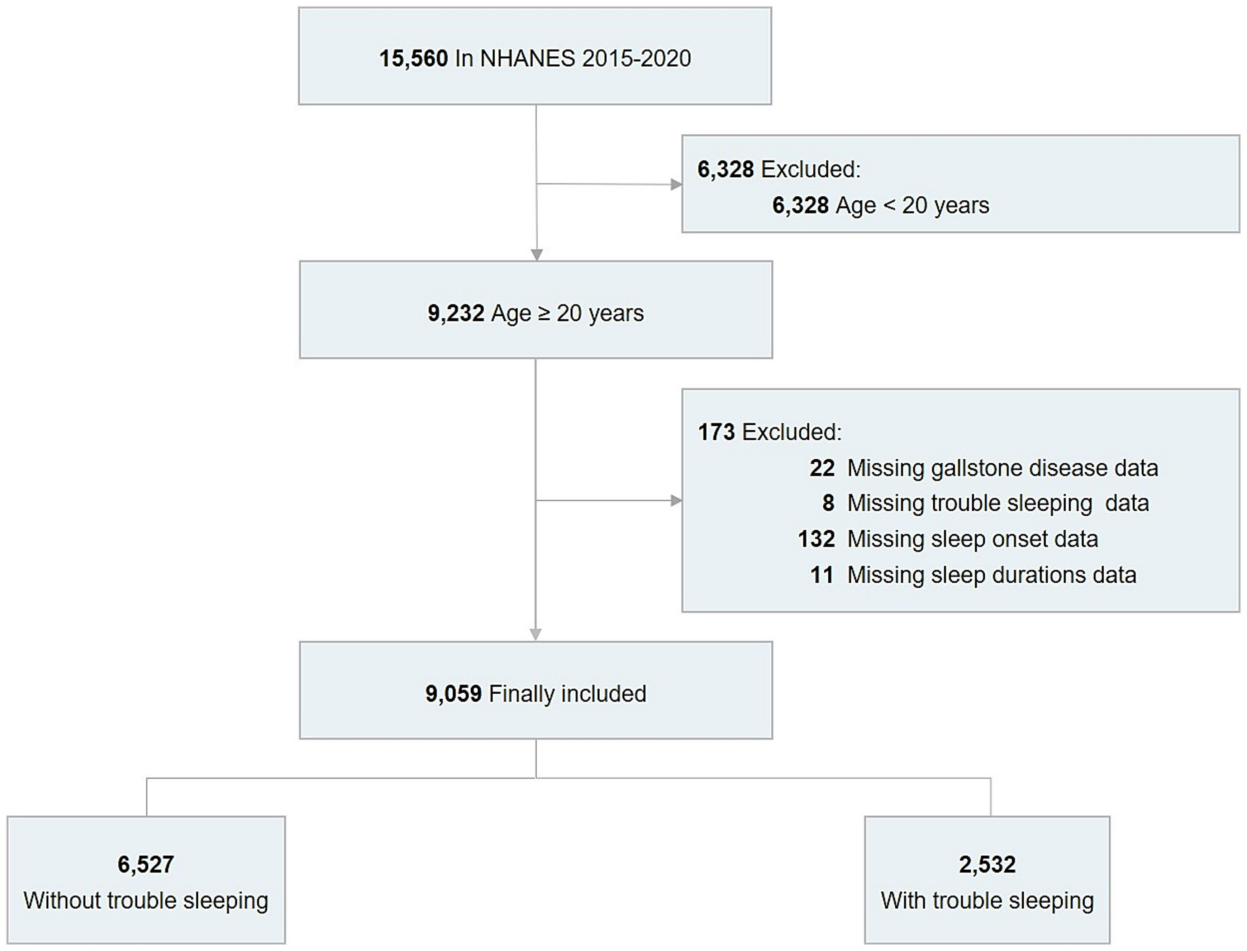
Flowchart of the screening process for the selection of eligible participants.

Data for this cross-sectional analysis were sourced from the National Health and Nutrition Examination Survey (NHANES) conducted between 2017 and 2020, initially comprising 15,560 participants. The exclusion criteria of this study included individuals under the age of 20, those with missing data on gallstone disease, and those lacking information about trouble sleeping, sleep onset time, and sleep duration (Supplementary methods). The study ultimately encompassed a cohort of 9,059 individuals (Figure 1).

The NHANES protocol received approval from the Institutional Review Board of the National Center for Health Statistics, and all participants provided written consent. Our analysis was exempt from Institutional Review Board approval under the Common Rule, as NHANES data are de-identified and publicly accessible. Additionally, this study followed the Strengthening the Reporting of Observational Studies in Epidemiology reporting guidelines.

### 2.2 Definition of outcome and exposure variables

Gallstone disease was defined based on a positive response to the inquiry, “Has a doctor or other health professional ever told you that you had gallstones?.” Trouble sleeping was identified as an affirmative response to the inquiry, “Have you ever told a doctor or other health professional that you have trouble sleeping?.” Sleep onset time was determined by the inquiry: “What time do you usually go to sleep on weekdays or workdays?.” Sleep duration was assessed by the question: “How much sleep do you usually get at night on weekdays or workdays?.” Sleep onset time was categorized into several intervals: [22:00–23:00], [23:00–00:00], [00:00–01:00], [01:00–20:00], and [20:00–22:00] based on their distribution. Sleep duration was grouped as < 6 h, 6 to 8 h, and ≥ 8 h. The sleep information was exclusively obtained from the NHANES questionnaire.

### 2.3 Covariates

The demographic variables included in the study were age (years), sex (male or female), race (Hispanic, non-Hispanic White, non-Hispanic Black, or others), the ratio of family income to poverty, education levels (high school diploma or less, some college, or bachelor’s degree or more), insurance status (private insurance, other insurance, or not covered any insurance), smoking status (never, former, current), drinking status (none, moderate, heavy), marital status (married, living with a partner, separated, divorced, widowed, or never married), and body mass index (BMI). Laboratory measures included total cholesterol (TC), high-density lipoprotein cholesterol (HDL-c), hemoglobin A1c (HbA1c), triglyceride (TG), albumin, total bilirubin (TBIL), alanine aminotransferase (ALT), high-sensitivity C-reactive protein (HS-CRP), and uric acid (UA), all sourced from NHANES laboratory files. Physical activity (PA) is classified as sedentary, moderate, and vigorous activities, and comorbidities such as nonalcoholic fatty liver disease (NAFLD), anemia, diabetes mellitus (DM), hypertension (HT), stroke, cardiovascular disease (CVD), and chronic kidney disease (CKD) were also considered. Further details on covariates definitions are available in Supplementary Table S1.

### 2.4 Statistical analysis

This study employed complex sample weighting analysis using the NHANES-recommended sample weights to account for the survey design. Continuous variables were reported as mean ± standard error (SE), while categorical variables were summarized by number and percentage. Differences in baseline characteristics among groups were assessed using the Student’s t-test for continuous variables, and the Chi-squared test was employed for categorical data. Variables showing statistically significant differences in the baseline comparison were included in further analyses. Multivariate logistic regression analysis was conducted to evaluate the association between trouble sleeping and gallstone disease. We built 3 models as follows: model 1 was adjusted for age and sex; model 2 was further adjusted for race, BMI, marital status, insurance status, smoking status, PA, sleep duration, and sleep onset time; model 3 additionally included TC, TG, ALT, HbA1c, HS-CRP, albumin, DM, CVD, HT, and stroke. The study population was further stratified into two subgroups to explore the associations of sleep onset time and duration with gallstone disease among individuals with or without trouble sleeping, respectively.

The statistical analyses were conducted utilizing R software (Version 4.3.1). The statistical significance of the findings was assessed using two-tailed tests, with a significance threshold set at *p* < 0.05.

## 3 Results

### 3.1 Baseline characteristics

In total, 9,059 participants were included. The average age was 48.37 ± 0.53 years, and 51.85% were females. Compared to those without trouble sleeping, individuals with trouble sleeping were inclined to be older, female, and non-Hispanic White. The rates of late sleep onset time and short sleep duration were significantly higher among individuals with trouble sleeping. Those with trouble sleeping were more likely to report current or former smoking, have higher BMI levels, and greater coverage of health insurance, engage in less vigorous physical activity, and be widowed, divorced, or separated. Individuals experiencing trouble sleeping exhibited higher levels of TG, TC, ALT, HbA1c, and HS-CRP, along with reduced albumin levels. Additionally, participants with trouble sleeping showed a higher prevalence of DM, HT, stroke, and CVD. Detailed information on participants’ baseline characteristics was provided in Table 1.

**Table 1:** Baseline characteristics of the study groups.

Characteristics	Total	Without gallstone disease	With gallstone disease	*p*-value
Age, mean ± SE, years	48.37 ± 0.53	47.13 ± 0.57	51.29 ± 0.59	< 0.001
Sex (%)				< 0.001
Female	51.85	49.52	57.33	
Male	48.15	50.48	42.67	
Race (%)				< 0.001
Hispanic	15.95	17.47	12.37	
Non-Hispanic Black	11.34	12.16	9.40	
Non-Hispanic White	62.68	59.63	69.85	
Other	10.04	10.74	8.38	
The ratio of family income to poverty (%)				0.7
<1.30	16.38	18.39	19.50	
1.30–2.99	24.66	28.22	28.14	
≥3.00	46.43	53.39	52.36	
Educational levels				0.09
Bachelor’s degree or more	31.73	32.85	29.16	
Some college	30.52	29.23	33.62	
High school diploma or less	37.68	37.91	37.22	
Marital status (%)				< 0.001
Married/living with partner	61.88	63.38	58.39	
Divorced/separated/widowed	18.67	16.18	24.55	
Never married	19.42	20.43	17.06	
Insurance status (%)				< 0.001
Not covered any insurance	13.03	15.19	8.03	
Other insurance	26.49	23.40	33.89	
Private insurance	60.31	61.41	58.07	
Drinking status (%)				0.84
None	63.30	73.61	73.93	
Moderate	6.75	8.04	7.45	
Heavy	15.83	18.35	18.62	
Smoking status (%)				< 0.001
Never	57.70	60.62	50.88	
Former	25.74	24.03	29.79	
Current	16.53	15.35	19.33	
PA (%)				< 0.001
Sedentary	24.47	24.06	25.44	
Moderate	28.53	26.94	32.26	
Vigorous	47.00	49.00	42.30	
BMI, mean ± SE, kg/m^2^	29.84 ± 0.18	29.14 ± 0.15	31.47 ± 0.32	< 0.001
Sleep duration (%)				< 0.001
6–8 h	44.40	45.66	41.42	
<6 h	8.13	6.70	11.50	
≥8 h	47.47	47.64	47.07	
Sleep onset time intervals (%)				< 0.001
[20:00–22:00]	17.01	17.32	16.29	
[22:00–23:00]	28.67	30.39	24.62	
[23:00–00:00]	27.32	27.58	26.70	
[00:00–01:00]	13.06	12.36	14.70	
[01:00–20:00]	13.95	12.36	17.68	
HbA1c, mean ± SE, %	5.68 ± 0.02	5.64 ± 0.02	5.78 ± 0.03	< 0.001
TC, mean ± SE, mmol/L	4.86 ± 0.03	4.83 ± 0.03	4.91 ± 0.04	0.02
HDL-c, mean± SE, mmol/L	1.39 ± 0.01	1.39 ± 0.01	1.39 ± 0.02	0.56
TG, mean ± SE, mmol/L	1.58 ± 0.03	1.55 ± 0.04	1.66 ± 0.04	0.02
Albumin, mean ± SE, g/dl	4.10 ± 0.01	4.11 ± 0.01	4.07 ± 0.02	0.02
TBIL, mean ± SE, μmol/L	8.00 ± 0.11	8.08 ± 0.13	7.83 ± 0.16	0.17
ALT, mean ± SE, U/L	22.70 ± 0.32	22.24 ± 0.35	23.73 ± 0.57	0.03
UA, mean ± SE, μmol/L	317.64 ± 1.58	316.64 ± 2.04	319.90 ± 1.80	0.22
HS-CRP, mean ± SE, mg/L	3.84 ± 0.13	3.61 ± 0.14	4.38 ± 0.23	0.01
CVD (%)	9.89	7.75	14.92	< 0.001
HT (%)	32.42	26.08	47.43	< 0.001
Stroke (%)	3.60	2.64	5.89	< 0.001
CKD (%)	13.44	14.94	16.71	0.13
Anemia (%)	6.28	6.83	7.53	0.45
DM (%)	12.82	10.67	17.89	< 0.001
NAFLD (%)	28.73	32.25	34.76	0.17

PA, physical activity; BMI, body mass index; HbA1c, hemoglobin A1c; TC, total cholesterol; HDL-c, high-density lipoprotein cholesterol; TG, triglyceride; TBIL, total bilirubin; ALT, alanine aminotransferase; UA, uric acid; HS-CRP, high-sensitivity C-reactive protein; CVD, cardiovascular disease; CKD, chronic kidney disease; DM, diabetes mellitus; HT, hypertension; NAFLD, nonalcoholic fatty liver disease.

### 3.2 Association of sleep disturbances with gallstone disease

The weighted percentage of participants with gallstone disease was 16.24% in those reporting trouble sleeping, compared to 8.59% in those without. Compared to individuals without trouble sleeping, those with trouble sleeping showed an association with an elevated risk of gallstone disease after controlling for sleep onset time and sleep duration, with an odds ratio (OR) of 1.47 (95% confidence interval [CI]: 1.01–2.15; Table 2).

**Table 2:** The association between trouble sleeping and gallstone disease.

Variable	Event, n (%)	Model 1		Model 2		Model 3	
		OR (95%)	*p*-value	OR (95%)	*p*-value	OR (95%)	*p*-value
Without trouble sleeping	560 (8.59)	ref		ref		ref	
With trouble sleeping	407 (16.24)	1.77 (1.28,2.45)	0.001	1.56 (1.02,2.38)	0.04	1.47 (1.01,2.15)	0.04

Model 1 adjusted for age and sex. Model 2 adjusted for age, sex, race, BMI, marital status, insurance status, smoking status, PA, sleep duration, and sleep onset time. Model 3 accounted for model 2 plus TC, TG, ALT, HbA1c, HS-CRP, albumin, DM, CVD, HT, and stroke.

**Figure 2:**
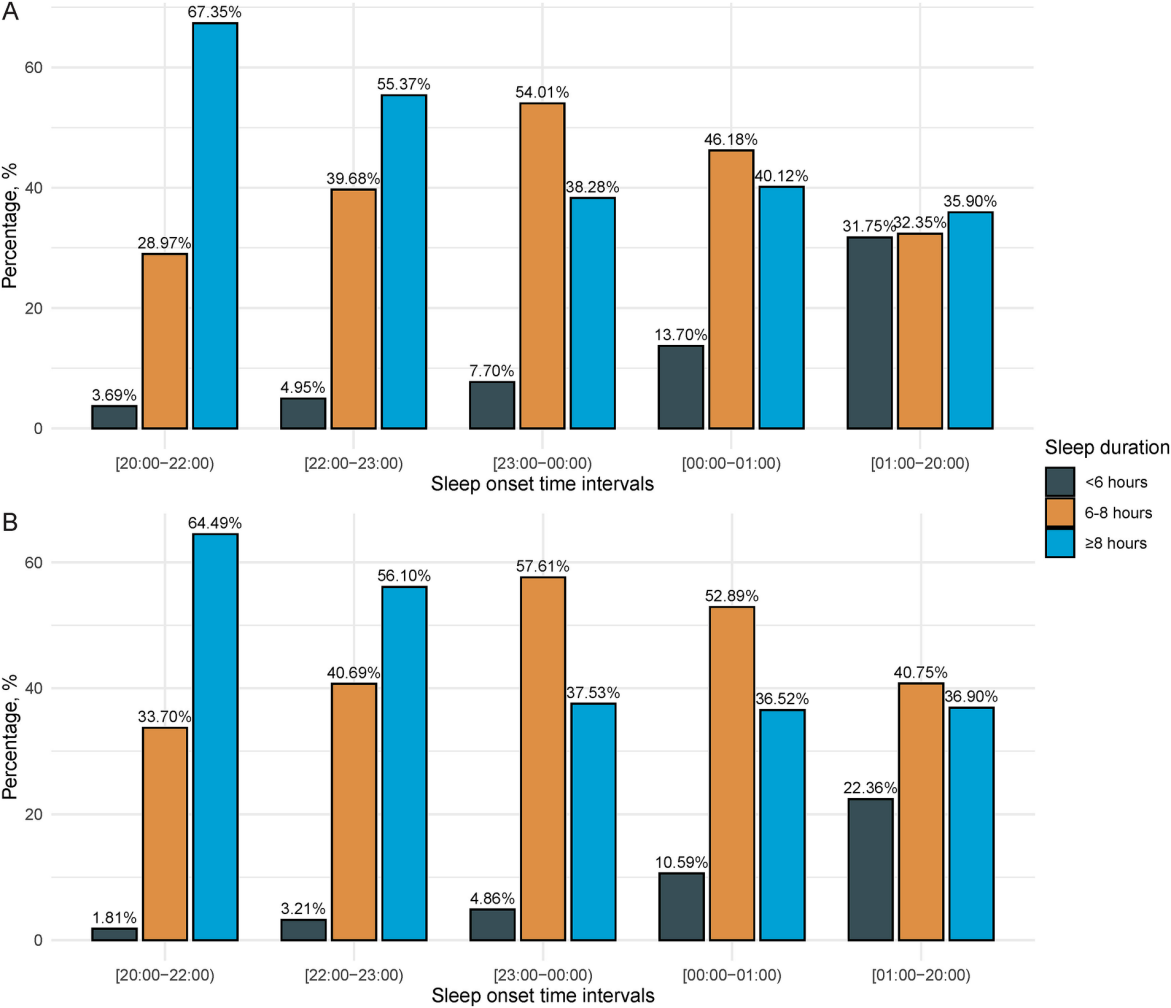
Distribution of sleep duration across sleep onset intervals in individuals with and without trouble sleeping. **(A)** Among those with trouble sleeping. **(B)** Among those without trouble sleeping.

Further stratified analysis was conducted according to the presence or absence of trouble sleeping. Individuals with earlier sleep onset time tended to experience long sleep duration (>8 h), whereas those with later sleep onset time tended to have a higher rate of short sleep duration (<6 h) among individuals with or without trouble sleeping. This relationship is visually depicted in Figure 2.

Among individuals with trouble sleeping, no significant associations were found between different sleep onset intervals and gallstone disease risk. The ORs were 1.03 (95% CI: 0.51–2.10) for 20:00–22:00, 0.78 (95% CI: 0.44–1.41) for 23:00–00:00, 1.22 (95% CI: 0.59–2.52) for 00:00–01:00, and 0.80 (95% CI: 0.46–1.40) for 01:00–20:00, relative to the reference sleep onset interval of 22:00–23:00. Likewise, no significant association was observed between sleep duration and gallstone disease. Relative to the reference sleep duration of 6–8 h, the ORs for sleep duration of <6 h and ≥8 h were 1.10 (95% CI: 0.63–1.92) and 0.70 (95% CI: 0.43–1.13), respectively (Table 3).

**Table 3:** The associations of sleep onset time and sleep duration with gallstone disease among individuals with trouble sleeping.

Variables	Event, n (%)	Model 1		Model 2		Model 3	
		OR (95%)	*p*-value	OR (95%)	*p*-value	OR (95%)	*p*-value
Sleep onset time intervals							
[22:00–23:00]	95 (16.49%)	ref		ref		ref	
[20:00–22:00]	59 (17.09%)	1.04 (0.51,2.12)	0.90	1.03 (0.45,2.36)	0.92	1.03 (0.51,2.10)	0.93
[23:00–00:00]	100 (14.26%)	0.95 (0.59,1.53)	0.82	0.82 (0.45,1.48)	0.43	0.78 (0.44,1.41)	0.40
[00:00–01:00]	71 (19.10%)	1.33 (0.68,2.63)	0.39	1.21 (0.52,2.80)	0.59	1.22 (0.59,2.52)	0.58
[01:00–20:00]	82 (15.74%)	1.09 (0.65,1.81)	0.73	0.85 (0.45,1.63)	0.55	0.80 (0.46,1.40)	0.41
Sleep duration							
6–8 h	150 (17.34%)	ref		ref		ref	
<6 h	68 (18.79%)	1.10 (0.72,1.69)	0.65	1.06 (0.55,2.02)	0.84	1.10 (0.63,1.92)	0.72
≥8 h	189 (14.66%)	0.69 (0.43,1.11)	0.12	0.68 (0.39,1.17)	0.13	0.70 (0.43,1.13)	0.14

Model 1 adjusted for age and sex. Model 2 adjusted for age, sex, race, BMI, marital status, insurance status, smoking status, PA, sleep duration, or sleep onset time. Model 3 accounted for model 2 plus TC, TG, ALT, HbA1c, HS-CRP, albumin, DM, CVD, HT, and stroke.

Conversely, among participants without trouble sleeping, the sleep onset interval of 23:00 to 00:00 was associated with a significantly increased risk of gallstone disease. The ORs were 0.91 (95% CI: 0.59–1.40) for 20:00–22:00, 1.61 (95% CI: 1.06–2.45) for 23:00–00:00, 1.05 (95% CI: 0.61–1.83) for 00:00–01:00, and 1.20 (95% CI: 0.75–1.92) for 01:00–20:00, relative to the reference group (22:00–23:00). Short sleep duration (less than 6 h) was associated with a significantly reduced risk of gallstone disease. Relative to the reference sleep duration of 6–8 h, the ORs were 0.43 (95% CI: 0.25–0.75) for < 6 h and 0.81 (95% CI: 0.57–1.15) for ≥ 8 h (Table 4).

**Table 4:** The associations of sleep onset time and sleep duration with gallstone disease among individuals without trouble sleeping.

Variables	Event, n (%)	Model 1		Model 2		Model 3	
		OR (95%)	*p*-value	OR (95%)	*p*-value	OR (95%)	*p*-value
Sleep onset time intervals							
[22:00–23:00]	146 (7.20%)	ref		ref		ref	
[20:00–22:00]	116 (8.80%)	1.18 (0.86,1.61)	0.28	1.05 (0.65,1.69)	0.82	0.91 (0.59,1.40)	0.66
[23:00–00:00]	166 (11.69%)	1.79 (1.25,2.58)	0.003	1.69 (1.03,2.77)	0.04	1.61 (1.06,2.45)	0.03
[00:00–01:00]	69 (7.09%)	1.16 (0.74,1.80)	0.50	1.13 (0.58,2.21)	0.65	1.05 (0.61,1.83)	0.84
[01:00–20:00]	63 (6.27%)	1.12 (0.72,1.75)	0.60	1.32 (0.76,2.31)	0.25	1.20 (0.75,1.92)	0.43
Sleep duration							
6–8 h	238 (8.99%)	ref		ref		ref	
<6 h	36 (4.56%)	0.48 (0.29,0.78)	0.005	0.47 (0.22,0.98)	0.05	0.43 (0.25,0.75)	0.004
≥8 h	286 (8.77%)	0.80 (0.58,1.09)	0.15	0.87 (0.56,1.34)	0.44	0.81 (0.57,1.15)	0.23

Model 1 adjusted for age and sex. Model 2 adjusted for age, sex, race, BMI, marital status, insurance status, smoking status, PA, sleep duration, or sleep onset time. Model 3 accounted for model 2 plus TC, TG, ALT, HbA1c, HS-CRP, albumin, DM, CVD, HT, and stroke.

## 4 Discussion

This cross-sectional study utilized extensive, nationally reflective data to elucidate the association between sleep disturbances and gallstone disease in American adults. Our findings suggested that individuals with trouble sleeping exhibited a 47% increased risk of gallstone disease, despite controlling for sleep onset time and duration. Notably, specific sleep timing, particularly onset from 23:00 to 00:00, was linked with a 61% elevated risk of gallstone disease among those without sleep disturbances, whereas short sleep duration (<6 h) was protective. These findings suggested that sleep timing and duration may interact differentially with gallstone disease risk based on the presence of sleep disturbances. This emphasized the potential of tailored sleep management strategies as a crucial component in the broader spectrum of gallstone disease prevention. Furthermore, it underscored the importance of addressing sleep disturbances in routine community health screening.

The first finding to emphasize is the significant association between trouble sleeping and increased risk of gallstone disease. There has been little research on the relationship between trouble sleeping and gallstone disease. However, a small volume of studies has explored the relationship between circadian rhythm disruption and the risk of gallstone disease. Trouble sleeping may represent a manifestation of circadian disorders (16, 17), and chronic trouble sleeping can eventually lead to circadian disruption (18). Both circadian disruption and trouble sleeping are associated with the development of various metabolic diseases (19–22). Given that gallstone disease is a consequence of metabolic dysregulation, it has also been linked to circadian disruption (5). He et al. conducted an animal study and found that circadian disorder may result in abnormal hepatic cholesterol and bile acid (BA) metabolism in mice, as a result promoting gallstone formation (14). Basnet et al. analyzed data from a population-based study involving the associations between chronotype and chronic diseases. They found that individuals with an evening activities preference, leading to circadian disruption, are associated with an elevated risk of gallstone disease (15). These studies provide supportive evidence for our findings. Our research addresses this gap by not only examining the relationship between trouble sleeping and gallstone disease but also exploring two additional dimensions of sleep: sleep onset time and sleep duration.

The next issue to discuss is the associations of sleep onset time and duration with the risk of gallstone disease among individuals with or without trouble sleeping. Long-term late sleep onset time and short sleep duration are often indicative of circadian rhythm disruption. Specifically, late sleep timing (e.g., between 23:00 and 00:00 or later) influenced by evening light exposure and voluntary behaviors, may lead to a delayed circadian phase and systematically shortened sleep duration (23). This occurs because wake time is constrained by work or school commitments, preventing parallel adjustments, and ultimately resulting in circadian misalignment (9, 24). Our research demonstrates that among individuals without trouble sleeping, a sleep onset time between 23:00 and 00:00 demonstrates an association with an increased risk of gallstone disease, whereas short sleep duration is inversely associated with this risk. However, individuals with sleep onset later than 23:00–00:00 did not exhibit an elevated risk of gallstone disease. Further investigation into the relationship between sleep onset time and sleep duration reveals that later bedtime often corresponds to shorter sleep duration. Notably, individuals with sleep onset time intervals after 23:00–00:00 exhibited a higher proportion of sleep duration less than 6 h.

The protective effect of short sleep duration may be attributed to bile excretion triggered by routine breakfast consumption after waking. This process may reduce bile storage time in the gallbladder and potentially limit the time available for cholesterol crystallization and gallstone formation. Additionally, among individuals with trouble sleeping, neither sleep onset time nor sleep duration was associated with gallstone disease. It is well-recognized that trouble sleeping often leads to delayed sleep onset and reduced total sleep duration (18). This discrepancy suggests that self-reported sleep onset time and sleep duration may not accurately reflect actual sleep patterns.

The underlying mechanisms linking sleep disturbances to gallstone formation remain unclear, though several hypotheses have been proposed. Sleep disturbances may represent a manifestation of circadian disorders. The link between circadian disruption and sleep disturbances is bidirectional, with prolonged sleep disturbances potentially leading to circadian disruption over time. Metabolic processes, including glucose, lipid, and endocrine regulation, are subject to circadian modulation (9). Circadian disruption has been implicated in the development of various metabolic diseases, which are recognized as risk factors for gallstone formation (5, 20). Over 90% of gallstones are primarily composed of cholesterol, commonly referred to as cholesterol gallstones (5). BA synthesis from cholesterol begins with cholesterol 7α-hydroxylase (Cyp7a1) in the canonical hepatocyte pathway. Following a meal, BA enter the duodenum, where gut microbiota metabolizes them throughout the gut (25). Circadian disruption may decrease gut microbial abundance, particularly bacteroidetes, thereby elevating bile salt hydrolase activity. This enhanced enzymatic activity increases the conversion of conjugated to free BA, which exhibit lower aqueous solubility. Consequently, these alterations reduce the bile acid pool. Consequently, these alterations reduce the bile acid pool. Circadian disruption may also suppress the circadian expression of core clock genes as well as other key genes, which may contribute to reduction of Cyp7a1 expression and reduced hepatic BAs synthesis (14, 26, 27). These changes could promote cholesterol saturation and crystallization in the gallbladder, thereby elevating the risk of cholesterol gallstone formation (5). Gallbladder hypomotility due to the extensive absorption of cholesterol by the gallbladder wall and chronic inflammation within the gallbladder wall may promote the formation of gallstones (5, 28, 29).

Based on our findings, we recommend that community health physicians recognize the role of sleep disturbances in gallstone formation, as addressing this factor could enhance primary prevention efforts. For individuals with trouble sleeping, interventions targeting circadian disruption may serve as adjunctive strategies to mitigate gallstone disease risk. Various circadian-based approaches have been developed to address circadian disorders, including morning light therapy, avoidance of evening light exposure, administration of exogenous melatonin or melatonin receptor agonists, and psychological and behavior interventions aimed at promoting adherence to treatment protocols (9, 30). An ideal treatment regimen may involve a combination of bright light therapy and melatonin supplementation (31). Accurately assessing the actual sleep patterns in individuals with trouble sleeping may assist in risk stratification for gallstone formation and facilitate the development of personalized intervention strategies.

The differential effects of sleep onset time and duration among individuals without trouble sleeping highlight the complexity of sleep patterns’ impact on metabolic and gallbladder health. The association between the sleep onset time interval of 23:00–00:00 and higher gallstone disease risk suggests that promoting earlier bedtime within this population might be beneficial. Furthermore, the protective effect of short sleep duration (< 6 h) on gallstone formation could be linked to reducing the time of bile stasis in the gallbladder. Individuals with sleep onset time later than midnight should be encouraged to adopt earlier bedtime habits. Prolonging sleep duration is not recommended; instead, waking up early to consume breakfast is advised to promote bile emptying from the gallbladder and reduce the risk of gallstone formation.

Although our study provided valuable insights, due to the cross-sectional nature of our analysis, it is impossible to establish a causal relationship based on the obtained results. Further longitudinal studies are required to confirm these findings and elucidate the temporal relationships between sleep disturbances and gallstone development. Although we adjusted for multiple known risk factors for gallstone disease, we acknowledge that certain unmeasured variables—such as dietary intake patterns and medication use (particularly lipid-lowering agents)—may also contribute to gallstone formation. Our study precludes the assessment of these factors, which represents an important limitation. Future prospective studies incorporating detailed dietary records and medication histories are needed to address this gap. Our study did not stratify the risk of gallstone disease according to specific subtypes of sleep disturbances, particularly failing to distinguish between individuals using hypnotic medications and those with primary sleep disorders (e.g., hypersomnia, sleep apnea). This limitation is clinically relevant, as pharmacological agents (e.g., benzodiazepines, Z-drugs) may modulate gallbladder motility and cholesterol metabolism through distinct mechanisms, potentially confounding the observed associations. Furthermore, we acknowledge an important limitation in the inability to apply Mendelian randomization to assess causal relationships. We recognize MR and other genetic epidemiological approaches as critical next steps for future research to substantiate these associations. Although our analysis adjusted for key metabolic confounders (e.g., BMI, DM, TC, and TG), we acknowledge the inability to incorporate eating time-related factors due to data unavailability in NHANES, which may represent an unmeasured confounding pathway between sleep quality and gallstone risk.

## 5 Conclusion

In conclusion, our study showed that trouble sleeping increased the risk of gallstone disease, independent of sleep onset time and sleep duration. Among those without trouble sleeping, a sleep onset time between 23:00–00:00 was associated with an elevated risk, while short sleep duration appeared protective. These findings highlighted the importance of considering sleep disturbances as a risk factor in the formation of gallstones. To substantiate these observations, further longitudinal studies and clinical trials are essential. These studies will help validate our findings and possibly develop targeted prevention strategies that incorporate sleep management as a core component.

## Data Availability

The raw data supporting the conclusions of this article will be made available by the authors, without undue reservation.
