# Supplementary material for: Associations of self-reported sleep disturbances, sleep onset, and duration with gallstone disease risk
Source: Front Nutr. 2025 Jun 24;12:1593720. doi: 10.3389/fnut.2025.1593720 (PMC12234331; doi:10.3389/fnut.2025.1593720)
Supplement: Supplementary file 1 [file Table_1.DOCX]

**Table S1.** The definitions of covariates.

| Covariables | Definitions |
| --- | --- |
| Physical activity |  |
| Vigorous | Work involves vigorous-intensity activity that causes large increases in breathing or heart rate like carrying or lifting heavy loads, digging or construction work for at least 10 minutes continuously |
| Moderate | Work involves moderate-intensity activity that causes small increases in breathing or heart rate such as brisk walking or carrying light loads for at least 10 minutes continuously |
| Sedentary | Sitting at school, at home, getting to and from places, or with friends including time spent sitting at a desk, traveling in a car or bus, reading, playing cards, watching television, or using a computer. Do not include time spent sleeping. |
| Cardiovascular disease | Doctor or other health professional told you had heart attack or doctor or other health professional told you had coronary heart disease or doctor or other health professional told you had angina or doctor or other health professional told you had congestive heart failure. |
| Stroke | Doctor or other health professional told you had a stroke. |
| Diabetes mellitus | Doctor or other health professional told you have diabetes or individuals had the administration of antidiabetic drugs |
| Hypertension | Doctor or other health professional told you had high blood pressure or an individual had mean measurements of systolic blood pressure ≥ 140 mmHg or mean measurements of diastolic blood pressure ≥ 90 mmHg or had the administration of antihypertensive medication |
| Chronic kidney disease | Urine albumin-creatinine ratio (uACR) equal to or more than 3mg/mmol or eGFR less than 60ml/min/1.73m^2^(1). |
| Anemia | Hemoglobin levels below 120g/L in women and below 130g/L in men. |
| Nonalcoholic fatty liver | We defined NAFLD by controlled attenuation parameter (CAP) scores of >=248 dB/m in absence of excessive alcohol use and viral hepatitis |
| Smoking status |  |
| Former | Participants who had smoked more than 100 cigarettes, but did not smoke at the time of survey |
| Current | Participants had smoked 100 cigarettes in their lifetime and smoked cigarettes at the time of survey |
| Never | Individuals who smoked less than 100 cigarettes in their lifetime |
| Drinking status |  |
| Heavy | Alcohol consumption was recorded at 28 grams per day for men and 14 grams per day for women |
| Moderate | Alcohol consumption was recorded ranging from 0.1 to 27.9 grams per day for men and 0.1 to 13.9 grams per day for women |
| None | Alcohol intake was recorded as 0 grams per day |

Reference

1. Kdigo 2021 Clinical Practice Guideline for the Management of Glomerular Diseases. *Kidney international* (2021) 100(4s):S1-s276. Epub 2021/09/25. doi: 10.1016/j.kint.2021.05.021.
