# Supplementary material for: Associations of self-reported sleep disturbances, sleep onset, and duration with gallstone disease risk
Source: Front Nutr. 2025 Jun 24;12:1593720. doi: 10.3389/fnut.2025.1593720 (PMC12234331; doi:10.3389/fnut.2025.1593720)
Supplement: Supplementary file 2 [file Table_2.DOCX]

**Direct HTML links for supporting and validating Figure 1 data**.

1. Excluded individuals age <20 years:

<https://wwwn.cdc.gov/Nchs/Data/Nhanes/Public/2017/DataFiles/P_DEMO.htm>

(2017-2020) survey

2. Excluded individuals missing sleep data:

<https://wwwn.cdc.gov/Nchs/Data/Nhanes/Public/2017/DataFiles/P_SLQ.htm>

(2017-2020) survey

3. Excluded individuals missing gallstone disease data:

<https://wwwn.cdc.gov/Nchs/Data/Nhanes/Public/2017/DataFiles/P_MCQ.htm#MCQ550>

(2017-2020) survey
